# Supplementary figures and images for: Mitochondrial genomics and phylogeny of noctuoid moths: Implications for Macroheterocera
Source: PLoS One. 2025 Oct 7;20(10):e0333540. doi: 10.1371/journal.pone.0333540 (PMC12503346; doi:10.1371/journal.pone.0333540)

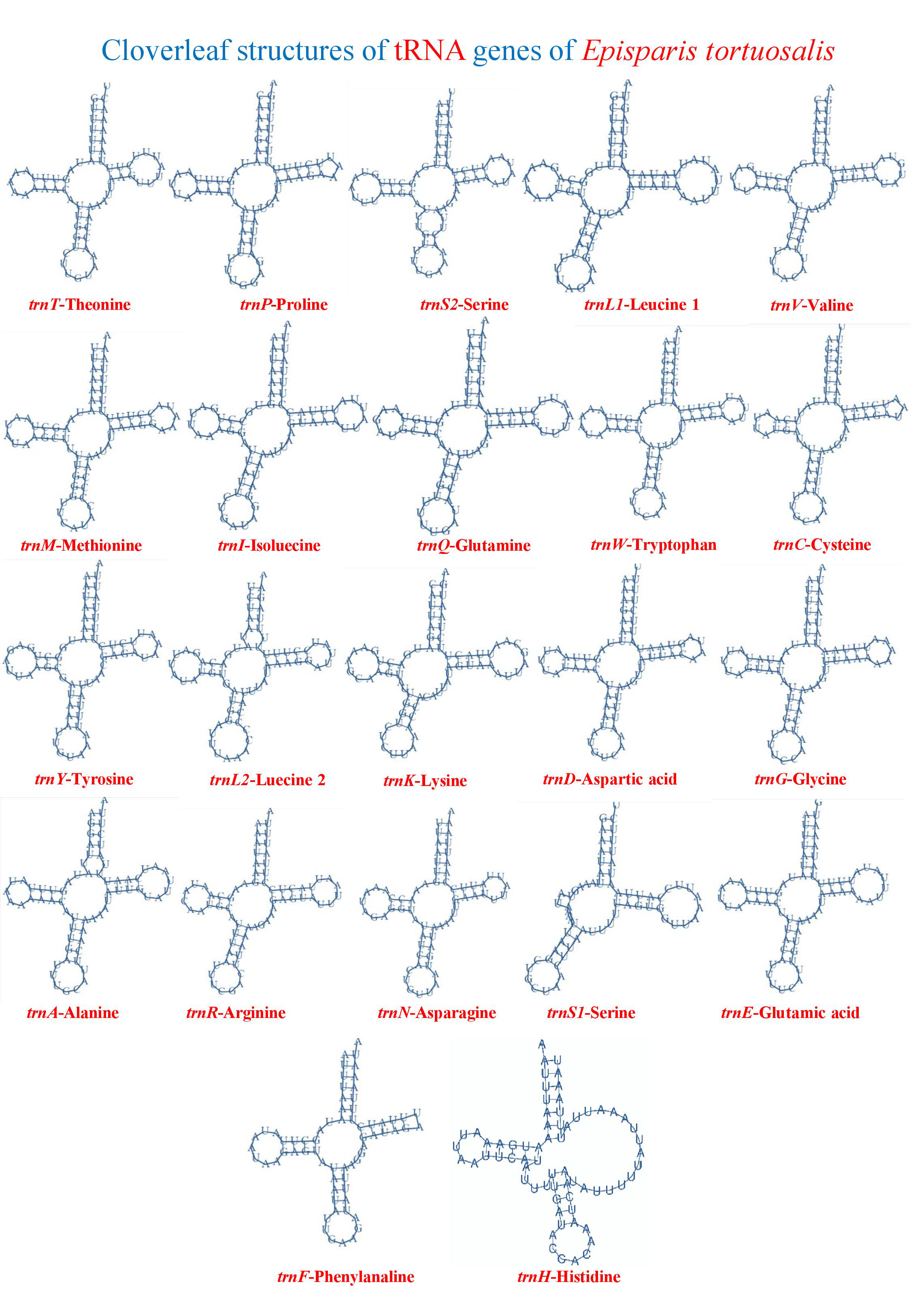

Supplement: S1 Fig — (TIF) [file pone.0333540.s001.tif]

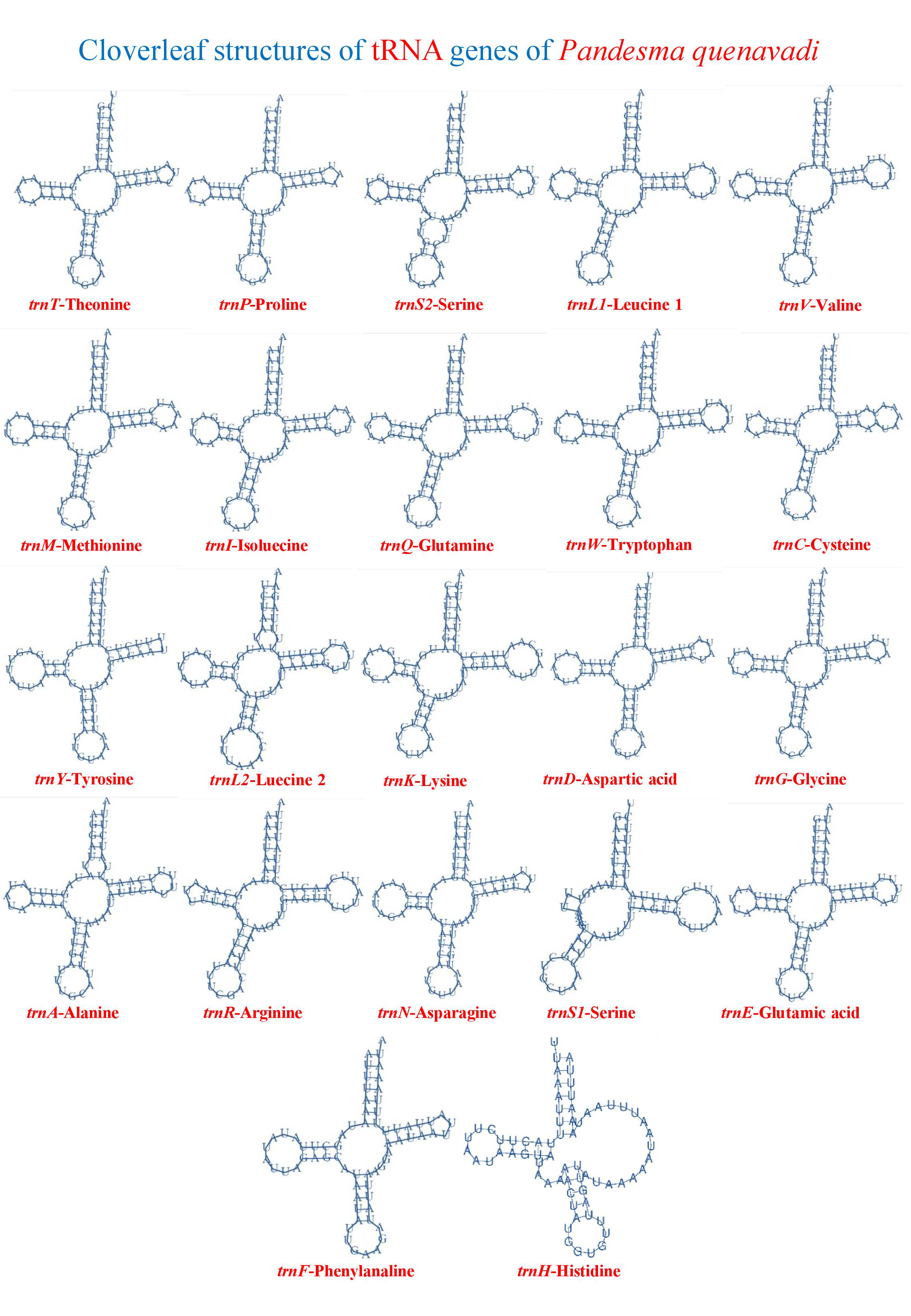

Supplement: S2 Fig — (TIF) [file pone.0333540.s002.tif]

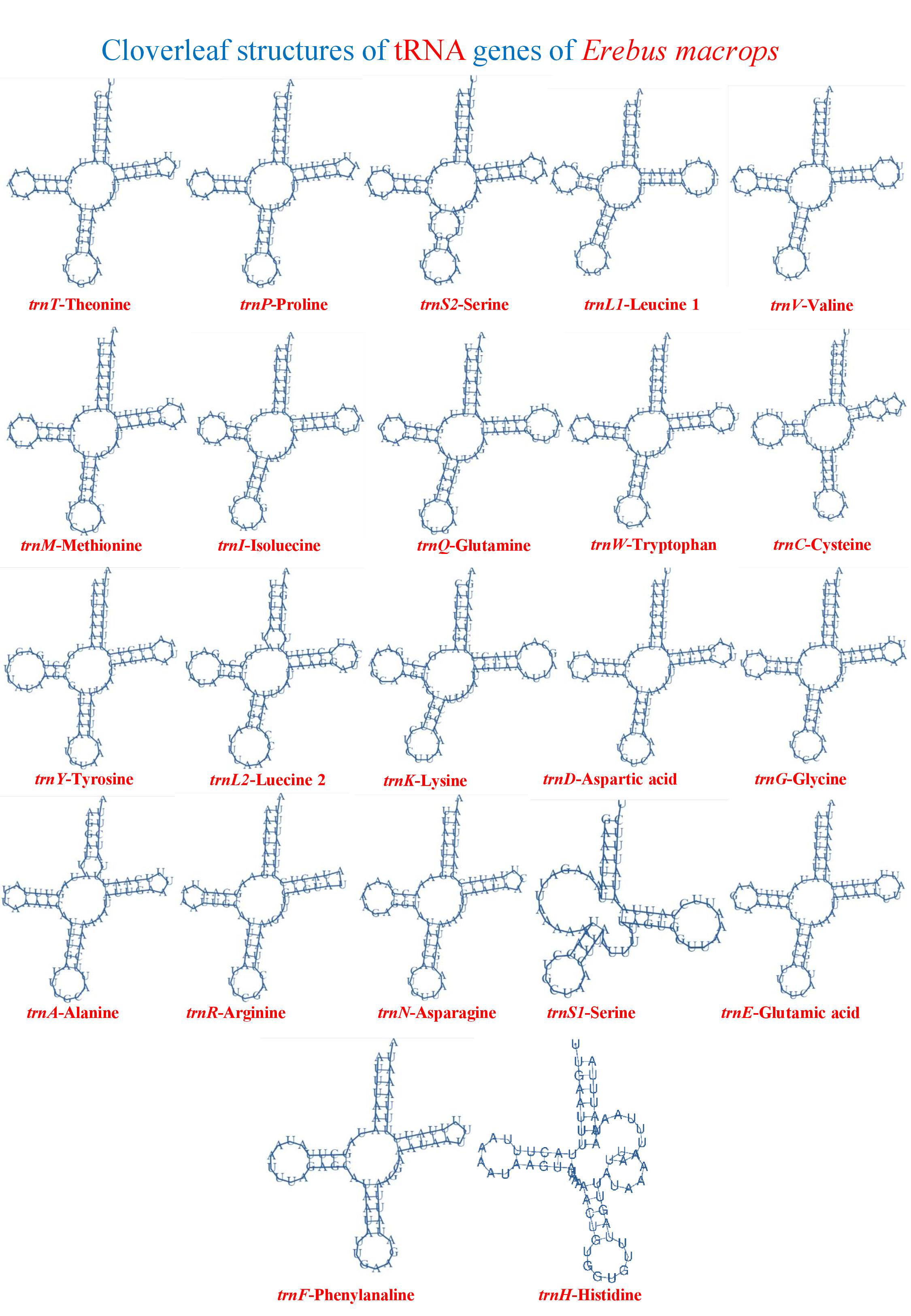

Supplement: S3 Fig — (TIF) [file pone.0333540.s003.tif]

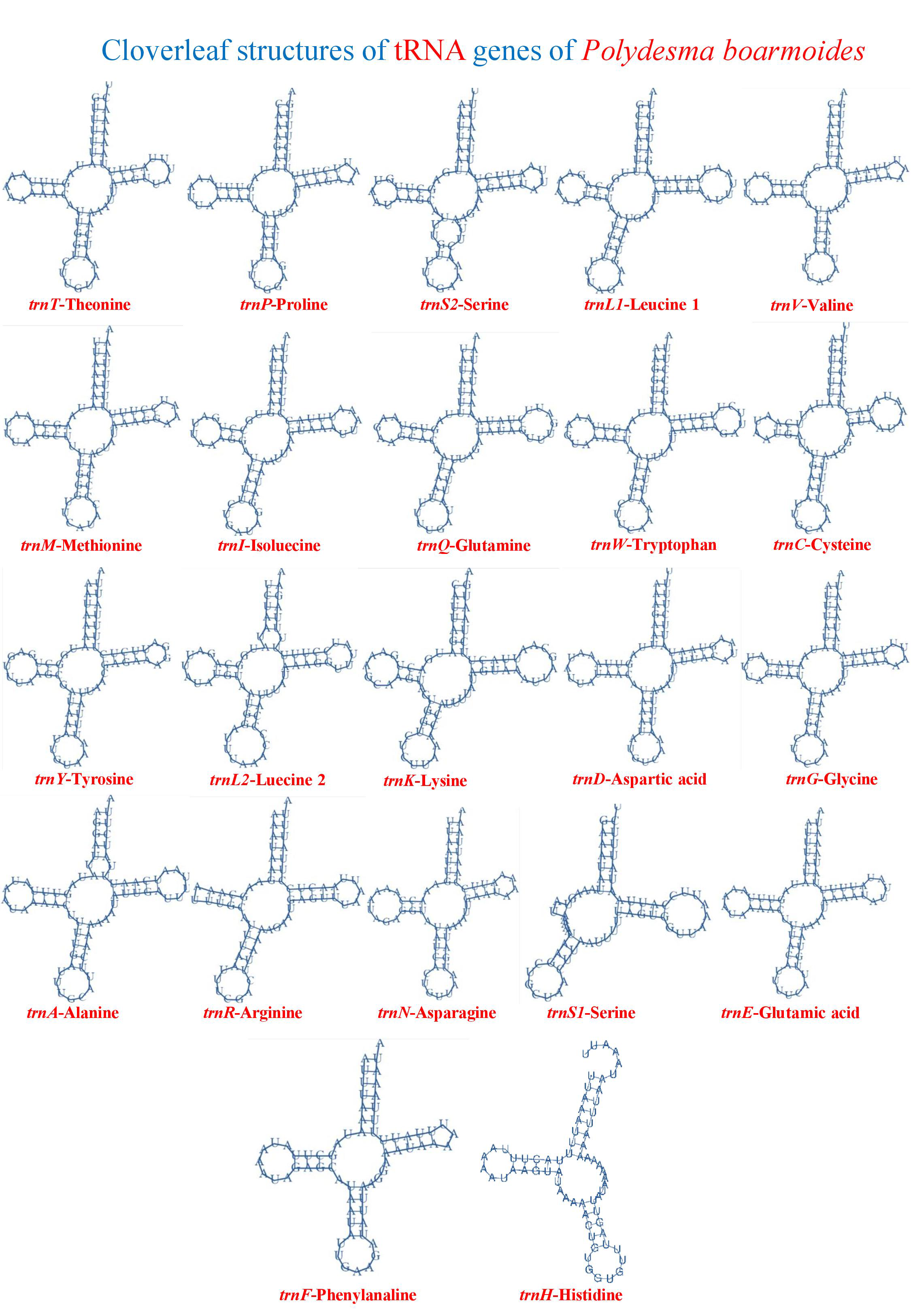

Supplement: S4 Fig — (TIF) [file pone.0333540.s004.tif]

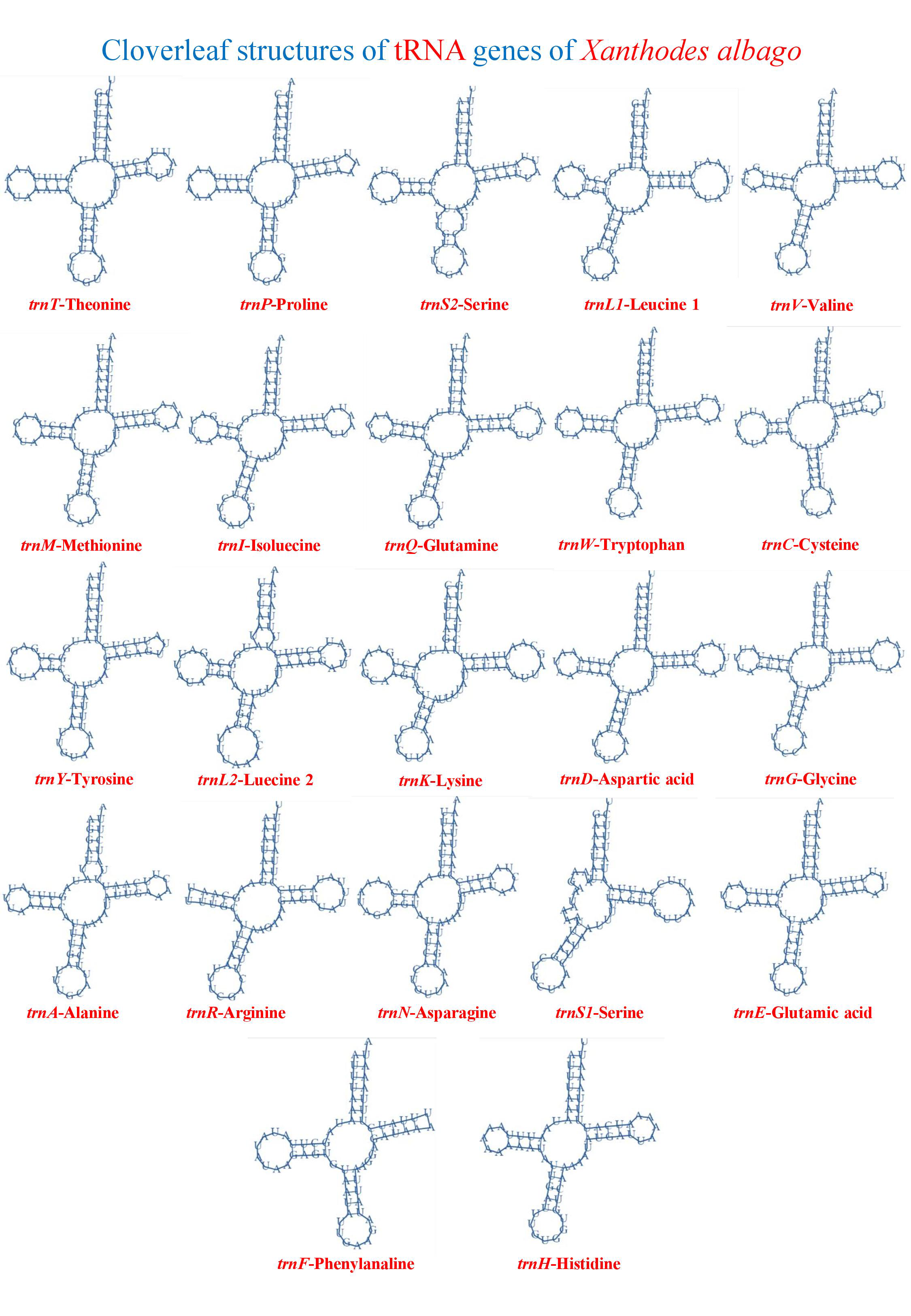

Supplement: S5 Fig — (TIF) [file pone.0333540.s005.tif]
