# Supplementary material for: Mitochondrial genomics and phylogeny of noctuoid moths: Implications for Macroheterocera
Source: PLoS One. 2025 Oct 7;20(10):e0333540. doi: 10.1371/journal.pone.0333540 (PMC12503346; doi:10.1371/journal.pone.0333540)
Supplement: S1 Table — (DOCX) [file pone.0333540.s006.docx]

**Supplementary Table 1**. List of complete mitogenomes of Macroheterocera superfamilies

| **Superfamily** | **Family** | **Subfamily** | **Species** | **Accession** |
| --- | --- | --- | --- | --- |
| Noctuoidea | Notodontidae | Phalerinae | *Phalera flavescens* | JF440342 |
|  |  | Thaumetopoeinae | *Ochrogaster lunifer* | AM946601 |
|  |  |  | *Thaumetopoea pityocampa* | MH286070 |
|  |  | Pygaerinae | *Clostera anachoreta* | KX108766 |
|  |  |  | *Clostera anastomosis* | MH286069 |
|  |  | Dudusinae | *Dudusa sphingiformis* | MW788876 |
|  |  | Stauropinae | *Neocerura liturata* | OK094456 |
|  |  | Cerurinae | *Syntypistis chambae* | MZ779034 |
|  |  | Notodontinae | *Peridea elzet* | OK235306 |
|  |  |  | *Pheosia rimosa* | OK149237 |
|  |  | Heterocampinae | *Cnethodonta grisescens* | MN956511 |
|  | Erebidae | Scoliopteryginae | *Anomis mesogona* | OK011995 |
|  |  |  | *Rusicada privata* | MW664368 |
|  |  | Calpinae | *Eudocima salaminia* | MW683337 |
|  |  |  | *Eudocima* *phalonia* | KY196412 |
|  |  |  | *Hemichloridia euprepia* | MW865754 |
|  |  | Toxocampinae | *Lygephila dorsigera* | MW648384 |
|  |  | Erebinae | *Catocala* sp. XY-2014 | KJ432280 |
|  |  |  | *Catocala electa* | MN698265 |
|  |  |  | *Hypospila bolinoides* | MW691121 |
|  |  |  | *Oraesia emarginata* | MW648382 |
|  |  |  | *Ophiusa tirhaca* | MW865752 |
|  |  |  | *Trigonodes hyppasia* | MW751988 |
|  |  |  | *Pandesma quenavadi* | **This study** |
|  |  |  | *Polydesma boarmoides* | **This study** |
|  |  |  | *Erebus macrops* | **This study** |
|  |  |  | *Erebus caprimulgus* | MZ964411 |
|  |  |  | *Sphingomorpha chlorea* | MW751990 |
|  |  |  | *Ercheia cyllaria* | MW751989 |
|  |  |  | *Grammodes geometrica* | KY888135 |
|  |  |  | *Chilkasa falcata* | MZ959073 |
|  |  |  | *Daddala lucilla* | MZ959069 |
|  |  |  | *Daddala brevicauda* | ON109239 |
|  |  |  | *Chrysopera combinans* | MZ964413 |
|  |  |  | *Calyptra minuticornis* | MZ944874 |
|  |  |  | *Corcobara angulipennis* | MW879210 |
|  |  |  | *Parallelia stuposa* | MK262707 |
|  |  |  | *Artena dotata* | MW697902 |
|  |  |  | *Mocis undata* | MW802208 |
|  |  |  | *Mocis ancilla* | OK149232 |

**Supplementary Table 1** continued

| **Superfamily** | **Family** | **Subfamily** | **Species** | **Accession** |
| --- | --- | --- | --- | --- |
|  |  |  | *Sympis rufibasis* | MW846302 |
|  |  |  | *Hulodes caranea* | OL335949 |
|  |  |  | *Lacera noctilio* | MW846301 |
|  |  |  | *Lacera procellosa* | ON109242 |
|  |  |  | *Thyas honesta* | MZ562561 |
|  |  |  | *Spirama retorta* | MT013356 |
|  |  |  | *Dysgonia illibata* | ON109240 |
|  |  |  | *Bastilla crameri* | ON109241 |
|  |  |  | *Singara diversalis* | OM933667 |
|  |  | *incertae sedis* | *Oxyodes scrobiculata* | MW865753 |
|  |  |  | *Ischyja manlia* | MW664367 |
|  |  |  | *Ischyja marapok* | ON165249 |
|  |  |  | *Cifuna locuples* | MK122622 |
|  |  |  | *Pida apicalis* | ON109243 |
|  |  | Aganainae | *Asota plana lacteata* | KJ173908 |
|  |  |  | *Asota paliura* | MZ944876 |
|  |  |  | *Asota caricae* | MZ779033 |
|  |  | Herminiinae | *Hydrillodes lentalis* | MH013484 |
|  |  |  | *Polypogon tarsicrinata* | MN635736 |
|  |  |  | *Simplicia niphona* | OK235310 |
|  |  |  | *Simplicia rectalis* | OK053110 |
|  |  | Hypeninae | *Paragabara curvicornuta* | KT362742 |
|  |  |  | *Colobochyla salicalis* | MK122621 |
|  |  | Arctiinae | *Hyphantria cunea* | GU592049 |
|  |  |  | *Asura megala* | MZ545409 |
|  |  |  | *Amerila alberti* | MZ927092 |
|  |  |  | *Phragmatobia fuliginosa* | OK094457 |
|  |  |  | *Amata formosae* | KC513737 |
|  |  |  | *Pareuchaetes insulata* | MZ964412 |
|  |  |  | *Vamuna virilis* | KJ364659 |
|  |  |  | *Callimorpha dominula* | KP973953 |
|  |  |  | *Nyctemera arctata albofasciata* | KM244681 |
|  |  |  | *Nyctemera adversata* | MZ562560 |
|  |  |  | *Paraona staudingeri* | KY827330 |
|  |  |  | *Spilosoma lubricipeda* | MT591568 |
|  |  |  | *Arctia plantaginis* | MW394229 |
|  |  |  | *Lemyra melli* | KP307017 |
|  |  |  | *Spilarctia subcarnea* | KT258909 |
|  |  |  | *Spilarctia casigneta* | MZ959068 |
|  |  |  | *Cyana* sp. MT-2014 | KM244679 |

**Supplementary Table 1** continued

| **Superfamily** | **Family** | **Subfamily** | **Species** | **Accession** |
| --- | --- | --- | --- | --- |
|  |  |  | *Eilema ussuricum* | MN696172 |
|  |  |  | *Aglaomorpha histrio* | KY800518 |
|  |  |  | *Brunia dorsalis* | MN635735 |
|  |  | Lymantriinae | *Lymantria dispar* | FJ617240 |
|  |  |  | *Lymantria sinica* | MZ087938 |
|  |  |  | *Lymantria mathura* | MZ073359 |
|  |  |  | *Lymantria dispar asiatica* | KY923067 |
|  |  |  | *Lymantria dispar japonica* | KY923060 |
|  |  |  | *Lymantria umbrosa* | KY923066 |
|  |  |  | *Lymantria sugii* | MT265380 |
|  |  |  | *Gynaephora minora* | KY688086 |
|  |  |  | *Gynaephora menyuanensis* | KC185412 |
|  |  |  | *Gynaephora jiuzhiensis* | KY688085 |
|  |  |  | *Gynaephora ruoergensis* | KY688083 |
|  |  |  | *Gynaephora qumalaiensis* | KJ507134 |
|  |  |  | *Gynaephora qinghaiensis* | KJ507133 |
|  |  |  | *Gynaephora rossii* | MW678846 |
|  |  |  | *Gynaephora aureata* | KY688084 |
|  |  |  | *Gynaephora groenlandica* | MW678847 |
|  |  |  | *Euproctis pseudoconspersa* | KJ716847 |
|  |  |  | *Euproctis similis* | KT258910 |
|  |  |  | *Euproctis cryptosticta* | KY996558 |
|  |  |  | *Euproctis seitzi* | MN916588 |
|  |  |  | *Somena scintillans* | MH051839 |
|  |  |  | *Lachana alpherakii* | KJ957168 |
|  |  |  | *Orgyia postica* | MW355619 |
|  |  |  | *Laelia suffusa* | MN908152 |
|  |  |  | *Laelia coenosa* | MK122630 |
|  |  |  | *Olene inclusa* | OK011992 |
|  |  |  | *Dasychira tristis* | MZ520324 |
|  |  |  | *Leucoma chrysoscela* | MW030505 |
|  |  |  | *Calliteara horishanella* | MZ571364 |
|  | Euteliidae | Euteliinae | *Eutelia adulatricoides* | KJ185131 |
|  |  |  | *Paectes cristatrix* | MW846303 |
|  |  | Stictopterinae | *Odontodes seranensis* | MW719565 |
|  | Noctuidae | Amphipyrinae | *Spodoptera exigua* | JX316220 |
|  |  |  | *Spodoptera litura* | KF543065 |
|  |  |  | *Spodoptera frugiperda* | KU877172 |
|  |  |  | *Spodoptera littoralis* | MT816470 |

**Supplementary Table 1** continued

| **Superfamily** | **Family** | **Subfamily** | **Species** | **Accession** |
| --- | --- | --- | --- | --- |
|  |  |  | *Spodoptera exempta* | MT906792 |
|  |  |  | *Spodoptera depravata* | OK053108 |
|  |  | Noctuinae | *Agrotis ipsilon* | KF163965 |
|  |  |  | *Agrotis tokionis* | MZ397981 |
|  |  |  | *Agrotis segetum* | KC894725 |
|  |  |  | *Agrotis munda* | MZ397914 |
|  |  |  | *Agrotis trifurca* | OK235311 |
|  |  |  | *Agrotis exclamationis* | MZ397982 |
|  |  |  | *Xestia c-nigrum* | MZ397980 |
|  |  |  | *Xestia dilatata* | MK122631 |
|  |  |  | *Noctua pronuba* | KJ508057 |
|  |  |  | *Striacosta albicosta* | KM488268 |
|  |  |  | *Actebia praecox* | MZ853167 |
|  |  |  | *Anaplectoides virens* | MZ707539 |
|  |  | Xyleninae | *Athetis lepigone* | MF152842 |
|  |  |  | *Athetis pallidipennis* | MT040606 |
|  |  |  | *Athetis thoracica* | MZ571409 |
|  |  |  | *Actinotia intermediata* | OK011997 |
|  |  |  | *Actinotia polyodon* | MW697903 |
|  |  |  | *Niphonyx segregata* | OK235315 |
|  |  |  | *Imosca coreana* | OK053111 |
|  |  | Heliothinae | *Helicoverpa zea* | KJ930516 |
|  |  |  | *Helicoverpa punctigera* | KF977797 |
|  |  |  | *Helicoverpa armigera* | GU188273 |
|  |  |  | *Helicoverpa assulta* | MG437198 |
|  |  |  | *Helicoverpa gelotopoeon* | MG437199 |
|  |  |  | *Chloridea subflexa* | KT598688 |
|  |  |  | *Pyrrhia umbra* | OK053112 |
|  |  | Acronictinae | *Acronicta psi* | KJ508060 |
|  |  |  | *Acronicta major* | MZ562565 |
|  |  |  | *Acronicta rumicis* | OK235313 |
|  |  | Hadeninae | *Mythimna separata* | KM099034 |
|  |  |  | *Mythimna turca* | MK122628 |
|  |  |  | *Mythimna pallidicosta* | MH027985 |
|  |  |  | *Mythimna loreyi* | MT506351 |
|  |  |  | *Leiometopon simyrides* | MW255962 |
|  |  |  | *Protegira songi* | KY379907 |
|  |  |  | *Mamestra configurata* | NDFZ01086780 |
|  |  |  | *Mamestra brassicae* | OK053109 |

**Supplementary Table 1** continued

| **Superfamily** | **Family** | **Subfamily** | **Species** | **Accession** |
| --- | --- | --- | --- | --- |
|  |  |  | *Anarta trifolii* | MN715147 |
|  |  |  | *Melanchra persicariae* | OK235309 |
|  |  |  | *Tiracola aureata* | OK011993 |
|  |  |  | *Tiracola plagiata* | MZ964414 |
|  |  | Plusiinae | *Ctenoplusia agnata* | KC414791 |
|  |  |  | *Ctenoplusia limbirena* | KM244665 |
|  |  |  | *Episparis tortuosalis* | **This study** |
|  |  |  | *Ctenoplusia albostriata* | MN495624 |
|  |  |  | *Trichoplusia ni* | MK714850 |
|  |  |  | *Macdunnoughia hybrida* | MW924384 |
|  |  |  | *Diachrysia nadeja* | MT916722 |
|  |  |  | *Abrostola triplasia* | OK235316 |
|  |  |  | *Chrysodeixis acuta* | OL892047 |
|  |  | Dyopsinae | *Arcte coerula* | MZ562562 |
|  |  | Cuculliinae | *Cucullia pustulata* | OK094455 |
|  |  | Ipimorphinae | *Cosmia restituta* | OK235307 |
|  |  | Condicinae | *Eucarta virgo* | OK235305 |
|  |  |  | *Condica illecta* | MZ562566 |
|  |  |  | *Condica capensis* | MZ571410 |
|  |  | Bagisarinae | *Xanthodes intersepta* | MZ571408 |
|  |  |  | *Xanthodes albago* | **This study** |
|  |  |  | *Sphragifera sigillata* | OK149231 |
|  | Nolidae | Nolinae | *Nola angustipennis* | ON462330 |
|  |  | Chloephorinae | *Gabala argentata* | KJ410747 |
|  |  |  | *Carea varipes* | ON939555 |
|  |  |  | *Sinna extrema* | MG872330 |
|  |  |  | *Camptoloma vanata* | MZ032042 |
|  |  |  | *Camptoloma kishidai* | MW435591 |
|  |  |  | *Pseudoips prasinana* | NC_062184 |
|  |  | Eariadinae | *Earias clorana* | OK235312 |
|  |  | Eligminae | *Eligma narcissus* | OK011994 |
|  |  | Risobinae | *Risoba prominens* | KJ396197 |
|  |  |  | *Risoba obstructa* | MW879211 |
| Lasiocampoidea | Lasiocampidae |  | *Trabala vishnou guttata* | KU884483 |
|  |  |  | *Euthrix laeta* | KU870700 |
|  |  |  | *Kunugia undans* | KX822016 |
|  |  |  | *Dendrolimus kikuchii* | NC_036347 |
| Bombycoidea | Sphingidae | Macroglossinae | *Macroglossum stellatarum* | MG747645 |
|  |  |  | *Dahira obliquifascia* | MZ343807 |

**Supplementary Table 1** continued

| **Superfamily** | **Family** | **Subfamily** | **Species** | **Accession** |
| --- | --- | --- | --- | --- |
|  |  |  | *Ampelophaga rubiginosa* | MT712133 |
|  |  |  | *Theretra japonica* | MG655620 |
|  |  |  | *Theretra oldenlandiae* | MN885801 |
|  |  | Sphinginae | *Psilogramma increta* | MF974243 |
|  |  |  | *Notonagemia analis scribae* | KU934302 |
|  |  |  | *Manduca sexta* | EU286785 |
|  |  |  | *Sphinx morio* | KC470083 |
|  |  | Smerinthinae | *Phyllosphingia dissimilis* | OL622044 |
|  |  |  | *Rhodoprasina callantha* | MZ343573 |
|  | Saturniidae | Saturniinae | *Actias selene* | JX186589 |
|  |  |  | *Antheraea pernyi* | MT890592 |
|  | Bombycidae | Bombycinae | *Bombyx mori* | AF149768 |
|  |  |  | *Bombyx mandarina* | MZ982840 |
| Geometroidea | Geometridae | Ennominae | *Biston panterinaria* | JX406146 |
|  |  |  | *Erannis ankeraria* | MN046105 |
|  |  |  | *Ectropis obliqua* | KX827002 |
|  |  |  | *Abraxas suspecta* | KY095828 |
|  |  |  | *Semiothisa cinerearia* | MK880228 |
|  |  |  | *Phthonandria atrilineata* | EU569764 |
|  |  |  | *Hypomecis punctinalis* | MK903031 |
|  |  |  | *Milionia basalis* | MN495623 |
|  |  | Larentiinae | *Pasiphila chloerata* | MN598218 |
|  |  |  | *Hydrelia parvulata* | MN962739 |
|  |  | Sterrhinae | *Idaea effusaria* | MN646772 |
|  |  | Geometrinae | *Iotaphora admirabilis* | MK903032 |
|  |  |  | *Lacanobia aliena* | MT040605 |
| Drepanoidea | Doidae |  | *Doa* sp. MJT-2014 | KJ508058 |
|  | Drepanidae | Drepaninae | *Drepana arcuata* | KJ508053 |
| **Outgroups** | Papilionoidea | Papilionidae | *Papilio polytes* | KM014701 |
|  |  |  | *Trogonoptera brookiana* | LT999986 |
