# Supplementary material for: Mitochondrial genomics and phylogeny of noctuoid moths: Implications for Macroheterocera
Source: PLoS One. 2025 Oct 7;20(10):e0333540. doi: 10.1371/journal.pone.0333540 (PMC12503346; doi:10.1371/journal.pone.0333540)
Supplement: S3 Table — (DOCX) [file pone.0333540.s008.docx]

**Supplementary Table 3.**

Relative synonymous codon usage of *Episparis tortuosalis*

| AAA(K) | 218.0 | 1.84 |
| --- | --- | --- |
| AAC(N) | 52.0 | 0.31 |
| AAG(K) | 19.0 | 0.16 |
| AAU(N) | 288.0 | 1.69 |
| ACA(T) | 64.0 | 1.54 |
| ACC(T) | 22.0 | 0.53 |
| ACG(T) | 10.0 | 0.24 |
| ACU(T) | 70.0 | 1.69 |
| AGA(R) | 55.0 | 3.14 |
| AGC(S) | 24.0 | 0.55 |
| AGG(R) | 17.0 | 0.97 |
| AGU(S) | 33.0 | 0.75 |
| AUA(I) | 232.0 | 1.06 |
| AUC(I) | 56.0 | 0.26 |
| AUG(M) | 23.0 | 1.00 |
| AUU(I) | 366.0 | 1.68 |
| CAA(Q) | 55.0 | 1.69 |
| CAC(H) | 8.0 | 0.20 |
| CAG(Q) | 10.0 | 0.31 |
| CAU(H) | 74.0 | 1.80 |
| CCA(P) | 51.0 | 1.96 |
| CCC(P) | 12.0 | 0.46 |
| CCG(P) | 1.0 | 0.04 |
| CCU(P) | 40.0 | 1.54 |
| CGA(R) | 20.0 | 1.14 |
| CGC(R) | 2.0 | 0.11 |
| CGG(R) | 1.0 | 0.06 |
| CGU(R) | 10.0 | 0.57 |
| CUA(L) | 29.0 | 0.39 |
| CUC(L) | 8.0 | 0.11 |
| CUG(L) | 8.0 | 0.11 |
| CUU(L) | 47.0 | 0.63 |
| GAA(E) | 54.0 | 1.74 |
| GAC(D) | 4.0 | 0.17 |
| GAG(E) | 8.0 | 0.26 |
| GAU(D) | 42.0 | 1.83 |
| GCA(A) | 31.0 | 1.63 |
| GCC(A) | 4.0 | 0.21 |
| GCG(A) | 0.0 | 0.00 |
| GCU(A) | 41.0 | 2.16 |
| GGA(G) | 87.0 | 2.97 |
| GGC(G) | 1.0 | 0.03 |
| GGG(G) | 2.0 | 0.07 |
| GGU(G) | 27.0 | 0.92 |
| GUA(V) | 53.0 | 2.19 |
| GUC(V) | 5.0 | 0.21 |
| GUG(V) | 4.0 | 0.16 |
| GUU(V) | 35.0 | 1.44 |
| UAA(*) | 204.0 | 2.10 |
| UAC(Y) | 40.0 | 0.37 |
| UAG(*) | 18.0 | 0.18 |
| UAU(Y) | 179.0 | 1.63 |
| UCA(S) | 88.0 | 2.00 |
| UCC(S) | 24.0 | 0.55 |
| UCG(S) | 3.0 | 0.07 |
| UCU(S) | 92.0 | 2.09 |
| UGA(W) | 70.0 | 0.72 |
| UGC(C) | 6.0 | 0.46 |
| UGG(W) | 5.0 | 1.00 |
| UGU(C) | 20.0 | 1.54 |
| UUA(L) | 330.0 | 4.43 |
| UUC(F) | 49.0 | 0.32 |
| UUG(L) | 25.0 | 0.34 |
| UUU(F) | 256.0 | 1.68 |

Relative synonymous codon usage of *Erebus macrops*

| AAA(K) | 219.0 | 1.55 |
| --- | --- | --- |
| AAC(N) | 90.0 | 0.50 |
| AAG(K) | 63.0 | 0.45 |
| AAU(N) | 273.0 | 1.50 |
| ACA(T) | 32.0 | 1.22 |
| ACC(T) | 32.0 | 1.22 |
| ACG(T) | 9.0 | 0.34 |
| ACU(T) | 36.0 | 1.37 |
| AGA(R) | 29.0 | 1.81 |
| AGC(S) | 38.0 | 1.06 |
| AGG(S) | 50.0 | 3.13 |
| AGU(S) | 26.0 | 0.73 |
| AUA(I) | 199.0 | 1.11 |
| AUC(I) | 99.0 | 0.55 |
| AUG(M) | 33.0 | 1.00 |
| AUU(I) | 242.0 | 1.34 |
| CAA(Q) | 63.0 | 1.40 |
| CAC(H) | 11.0 | 0.28 |
| CAG(Q) | 27.0 | 0.60 |
| CAU(H) | 68.0 | 1.72 |
| CCA(P) | 29.0 | 1.21 |
| CCC(P) | 32.0 | 1.33 |
| CCG(P) | 8.0 | 0.33 |
| CCU(P) | 27.0 | 1.13 |
| CGA(R) | 7.0 | 0.44 |
| CGC(R) | 1.0 | 0.06 |
| CGG(R) | 4.0 | 0.25 |
| CGU(R) | 5.0 | 0.31 |
| CUA(L) | 72.0 | 0.78 |
| CUC(L) | 40.0 | 0.43 |
| CUG(L) | 20.0 | 0.22 |
| CUU(L) | 53.0 | 0.57 |
| GAA(E) | 40.0 | 1.48 |
| GAC(D) | 9.0 | 0.53 |
| GAG(E) | 14.0 | 0.52 |
| GAU(D) | 25.0 | 1.47 |
| GCA(A) | 11.0 | 1.63 |
| GCC(A) | 3.0 | 0.44 |
| GCG(A) | 3.0 | 0.44 |
| GCU(A) | 10.0 | 1.48 |
| GGA(G) | 24.0 | 2.46 |
| GGC(G) | 2.0 | 0.21 |
| GGG(G) | 5.0 | 0.51 |
| GGU(G) | 8.0 | 0.82 |
| GUA(V) | 30.0 | 1.74 |
| GUC(V) | 8.0 | 0.46 |
| GUG(V) | 4.0 | 0.23 |
| GUU(V) | 27.0 | 1.57 |
| UAA(*) | 220.0 | 2.07 |
| UAC(Y) | 50.0 | 0.37 |
| UAG(*) | 50.0 | 0.47 |
| UAU(Y) | 218.0 | 1.63 |
| UCA(S) | 58.0 | 1.62 |
| UCC(S) | 34.0 | 0.95 |
| UCG(S) | 11.0 | 0.31 |
| UCU(S) | 48.0 | 1.34 |
| UGA(W) | 49.0 | 0.46 |
| UGC(C) | 22.0 | 0.98 |
| UGG(W) | 32.0 | 1.00 |
| UGU(C) | 23.0 | 1.02 |
| UUA(L) | 311.0 | 3.37 |
| UUC(F) | 111.0 | 0.56 |
| UUG(L) | 58.0 | 0.63 |
| UUU(F) | 286.0 | 1.44 |

Relative synonymous codon usage of *Pandesma quenavadi*

| AAA(K) | 209.0 | 1.80 |
| --- | --- | --- |
| AAC(N) | 79.0 | 0.40 |
| AAG(K) | 23.0 | 0.20 |
| AAU(N) | 314.0 | 1.60 |
| ACA(T) | 48.0 | 1.33 |
| ACC(T) | 43.0 | 1.19 |
| ACG(T) | 8.0 | 0.22 |
| ACU(T) | 45.0 | 1.25 |
| AGA(R) | 49.0 | 2.41 |
| AGC(R) | 41.0 | 0.71 |
| AGG(S) | 49.0 | 2.41 |
| AGU(S) | 47.0 | 0.82 |
| AUA(I) | 196.0 | 1.01 |
| AUC(I) | 83.0 | 0.43 |
| AUG(M) | 32.0 | 1.00 |
| AUU(I) | 304.0 | 1.56 |
| CAA(Q) | 43.0 | 1.83 |
| CAC(H) | 8.0 | 0.31 |
| CAG(Q) | 4.0 | 0.17 |
| CAU(H) | 43.0 | 1.69 |
| CCA(P) | 22.0 | 1.22 |
| CCC(P) | 21.0 | 1.17 |
| CCG(P) | 0.0 | 0.00 |
| CCU(P) | 29.0 | 1.61 |
| CGA(R) | 15.0 | 0.74 |
| CGC(R) | 1.0 | 0.05 |
| CGG(R) | 1.0 | 0.05 |
| CGU(R) | 7.0 | 0.34 |
| CUA(L) | 33.0 | 0.53 |
| CUC(L) | 9.0 | 0.14 |
| CUG(L) | 14.0 | 0.23 |
| CUU(L) | 53.0 | 0.85 |
| GAA(E) | 33.0 | 1.69 |
| GAC(D) | 3.0 | 0.21 |
| GAG(E) | 6.0 | 0.31 |
| GAU(D) | 26.0 | 1.79 |
| GCA(A) | 12.0 | 1.04 |
| GCC(A) | 1.0 | 0.09 |
| GCG(A) | 0.0 | 0.00 |
| GCU(A) | 33.0 | 2.87 |
| GGA(G) | 49.0 | 2.93 |
| GGC(G) | 0.0 | 0.00 |
| GGG(G) | 1.0 | 0.06 |
| GGU(G) | 17.0 | 1.01 |
| GUA(V) | 34.0 | 2.43 |
| GUC(V) | 1.0 | 0.07 |
| GUG(V) | 1.0 | 0.07 |
| GUU(V) | 20.0 | 1.43 |
| UAA(*) | 265.0 | 2.18 |
| UAC(Y) | 57.0 | 0.37 |
| UAG(*) | 21.0 | 0.17 |
| UAU(Y) | 252.0 | 1.63 |
| UCA(S) | 92.0 | 1.60 |
| UCC(S) | 55.0 | 0.96 |
| UCG(S) | 21.0 | 0.37 |
| UCU(S) | 89.0 | 1.55 |
| UGA(*) | 78.0 | 0.64 |
| UGC(C) | 26.0 | 0.85 |
| UGG(W) | 27.0 | 1.00 |
| UGU(C) | 35.0 | 1.15 |
| UUA(L) | 226.0 | 3.64 |
| UUC(F) | 83.0 | 0.49 |
| UUG(L) | 38.0 | 0.61 |
| UUU(F) | 257.0 | 1.51 |

Relative synonymous codon usage of *Polydesma boarmoides*

| AAA(K) | 216.0 | 1.64 |
| --- | --- | --- |
| AAC(N) | 73.0 | 0.44 |
| AAG(K) | 47.0 | 0.36 |
| AAU(N) | 257.0 | 1.56 |
| ACA(T) | 44.0 | 1.38 |
| ACC(T) | 34.0 | 1.06 |
| ACG(T) | 10.0 | 0.31 |
| ACU(T) | 40.0 | 1.25 |
| AGA(S) | 46.0 | 2.88 |
| AGC(S) | 27.0 | 0.61 |
| AGG(S) | 31.0 | 1.94 |
| AGU(S) | 35.0 | 0.79 |
| AUA(M) | 214.0 | 1.16 |
| AUC(I) | 84.0 | 0.45 |
| AUG(M) | 30.0 | 1.00 |
| AUU(I) | 256.0 | 1.39 |
| CAA(Q) | 74.0 | 1.48 |
| CAC(H) | 16.0 | 0.42 |
| CAG(Q) | 26.0 | 0.52 |
| CAU(H) | 60.0 | 1.58 |
| CCA(P) | 22.0 | 1.14 |
| CCC(P) | 24.0 | 1.25 |
| CCG(P) | 2.0 | 0.10 |
| CCU(P) | 29.0 | 1.51 |
| CGA(R) | 11.0 | 0.69 |
| CGC(R) | 2.0 | 0.13 |
| CGG(R) | 1.0 | 0.06 |
| CGU(R) | 5.0 | 0.31 |
| CUA(L) | 64.0 | 0.77 |
| CUC(L) | 29.0 | 0.35 |
| CUG(L) | 19.0 | 0.23 |
| CUU(L) | 60.0 | 0.73 |
| GAA(E) | 45.0 | 1.41 |
| GAC(D) | 14.0 | 0.54 |
| GAG(E) | 19.0 | 0.59 |
| GAU(D) | 38.0 | 1.46 |
| GCA(A) | 19.0 | 1.69 |
| GCC(A) | 4.0 | 0.36 |
| GCG(A) | 0.0 | 0.00 |
| GCU(A) | 22.0 | 1.96 |
| GGA(G) | 46.0 | 3.23 |
| GGC(G) | 0.0 | 0.00 |
| GGG(G) | 0.0 | 0.00 |
| GGU(G) | 11.0 | 0.77 |
| GUA(V) | 32.0 | 1.83 |
| GUC(V) | 11.0 | 0.63 |
| GUG(V) | 6.0 | 0.34 |
| GUU(V) | 21.0 | 1.20 |
| UAA(*) | 263.0 | 2.19 |
| UAC(Y) | 46.0 | 0.33 |
| UAG(*) | 45.0 | 0.38 |
| UAU(Y) | 229.0 | 1.67 |
| UCA(S) | 64.0 | 1.44 |
| UCC(S) | 45.0 | 1.01 |
| UCG(S) | 13.0 | 0.29 |
| UCU(S) | 83.0 | 1.87 |
| UGA(*) | 52.0 | 0.43 |
| UGC(C) | 17.0 | 0.97 |
| UGG(W) | 16.0 | 1.00 |
| UGU(C) | 18.0 | 1.03 |
| UUA(L) | 269.0 | 3.25 |
| UUC(F) | 99.0 | 0.58 |
| UUG(L) | 55.0 | 0.67 |
| UUU(F) | 245.0 | 1.42 |

Relative synonymous codon usage of *Xanthodes albago*

| AAA(K) | 235.0 | 1.75 |
| --- | --- | --- |
| AAC(N) | 58.0 | 0.31 |
| AAG(K) | 34.0 | 0.25 |
| AAU(N) | 316.0 | 1.69 |
| ACA(T) | 64.0 | 1.64 |
| ACC(T) | 26.0 | 0.67 |
| ACG(T) | 6.0 | 0.15 |
| ACU(T) | 60.0 | 1.54 |
| AGA(R) | 48.0 | 2.74 |
| AGC(S) | 24.0 | 0.54 |
| AGG(R) | 25.0 | 1.43 |
| AGU(S) | 34.0 | 0.76 |
| AUA(I) | 231.0 | 1.09 |
| AUC(I) | 46.0 | 0.22 |
| AUG(M) | 19.0 | 1.00 |
| AUU(I) | 358.0 | 1.69 |
| CAA(Q) | 61.0 | 1.63 |
| CAC(H) | 6.0 | 0.19 |
| CAG(Q) | 14.0 | 0.37 |
| CAU(H) | 56.0 | 1.81 |
| CCA(P) | 45.0 | 1.75 |
| CCC(P) | 10.0 | 0.39 |
| CCG(P) | 0.0 | 0.00 |
| CCU(P) | 48.0 | 1.86 |
| CGA(R) | 23.0 | 1.31 |
| CGC(R) | 0.0 | 0.00 |
| CGG(R) | 0.0 | 0.00 |
| CGU(R) | 9.0 | 0.51 |
| CUA(L) | 47.0 | 0.64 |
| CUC(L) | 13.0 | 0.18 |
| CUG(L) | 6.0 | 0.08 |
| CUU(L) | 36.0 | 0.49 |
| GAA(E) | 61.0 | 1.91 |
| GAC(D) | 6.0 | 0.25 |
| GAG(E) | 3.0 | 0.09 |
| GAU(D) | 42.0 | 1.75 |
| GCA(A) | 22.0 | 1.24 |
| GCC(A) | 2.0 | 0.11 |
| GCG(A) | 0.0 | 0.00 |
| GCU(A) | 47.0 | 2.65 |
| GGA(G) | 84.0 | 3.03 |
| GGC(G) | 1.0 | 0.04 |
| GGG(G) | 2.0 | 0.07 |
| GGU(G) | 24.0 | 0.86 |
| GUA(V) | 44.0 | 2.44 |
| GUC(V) | 3.0 | 0.17 |
| GUG(V) | 1.0 | 0.06 |
| GUU(V) | 24.0 | 1.33 |
| UAA(*) | 151.0 | 1.83 |
| UAC(Y) | 33.0 | 0.30 |
| UAG(*) | 26.0 | 0.32 |
| UAU(Y) | 187.0 | 1.70 |
| UCA(S) | 104.0 | 2.32 |
| UCC(S) | 34.0 | 0.76 |
| UCG(S) | 10.0 | 0.22 |
| UCU(S) | 63.0 | 1.41 |
| UGA(*) | 70.0 | 0.85 |
| UGC(C) | 8.0 | 0.50 |
| UGG(W) | 14.0 | 1.00 |
| UGU(C) | 24.0 | 1.50 |
| UUA(L) | 309.0 | 4.22 |
| UUC(F) | 64.0 | 0.36 |
| UUG(L) | 28.0 | 0.38 |
| UUU(F) | 287.0 | 1.64 |
