# Supplementary material for: Mitochondrial genomics and phylogeny of noctuoid moths: Implications for Macroheterocera
Source: PLoS One. 2025 Oct 7;20(10):e0333540. doi: 10.1371/journal.pone.0333540 (PMC12503346; doi:10.1371/journal.pone.0333540)
Supplement: Attachment — Submitted filename: PONE-D-24-54093.docx [file pone.0333540.s009.docx]

**Manuscript PONE-D-24-54093**

I have thoroughly reviewed your manuscript titled *"Evolutionary and Phylogenetic Insights from the Mitochondrial Genomic Analyses of Noctuoid Moths (Lepidoptera: Noctuoidea) and Their Implications for the Macroheterocera Clade,"* and found it to be quite intriguing. I would like to share my suggestions and comments on the current draft, which I believe could serve as helpful guidelines for enhancing the overall quality and clarity of your work.

**Comments:**

**Title:**

The title is clear and accurately reflects the focus of the research, which appears to be the mitochondrial genomic analysis of noctuoid moths and its implications for understanding the Macroheterocera clade. However, it might be slightly verbose. Simplifying the title without losing specificity could enhance readability. For instance, "Mitochondrial Genomics and Phylogeny of Noctuoid Moths: Implications for Macroheterocera" might be more concise.

**Abstract:**

The abstract provides a clear summary of the study's objectives, methods, and results. However, it could be more concise, particularly the discussion on the "ATN" start codon, which may be technical for some readers. Additionally, the research gap or significance of the study could be highlighted more explicitly. A brief mention of the specific software or models used in the phylogenetic analysis would also enhance transparency.

**Introduction:**

The introduction provides a comprehensive background on the Lepidoptera order, particularly focusing on the Macroheterocera clade, and sets up the research well. However, there are a few points that could be improved. First, the introduction could benefit from a clearer statement of the research gap or the specific objective of this study. While the background on the phylogenetic uncertainties within the Macroheterocera clade is informative, the introduction could more explicitly outline how this study addresses these uncertainties or contributes to resolving them. The discussion of previous studies is thorough but could be better organized, as some references to earlier work (e.g., regarding the phylogenetic position of Mimallonoidea and Drepanoidea) appear scattered. A more focused synthesis of past research could improve the flow and clarity. Additionally, while the details on the mitochondrial genome are relevant, some of the technical aspects, such as the role of the A+T-rich region, might be more succinctly explained. Lastly, the mention of the specific species studied is helpful, but it would be more impactful to briefly highlight why these species were chosen for this particular analysis and what their phylogenetic relevance is.

**Materials and Methods:**

The materials and methods section is generally well-structured, providing a clear overview of the experimental design and procedures used. However, the inclusion of lengthy details such as the full list of species and sequencing methods in the main text can overwhelm the reader. I recommend moving the detailed table (Table 1) into a supplementary file for better clarity and ease of reading. Additionally, while the methods for genomic DNA extraction, sequencing, and annotation are described in detail, further justification for the choice of specific software and tools (such as NOVOPLASTY and MITOS2) would enhance the reproducibility of the study. Consider adding brief explanations on why these tools were selected over others in the context of this study. Finally, the phylogenetic analysis methods are appropriately outlined, though a clearer explanation of how the outgroup species were chosen could help contextualize the analysis further.

**Results and Discussion:**

The **Results** and **Discussion** sections are well-structured and clear, effectively presenting the study's findings and their implications. The results are supported by solid statistical analyses, but further clarification on the sampling and data collection methods would strengthen the validity of the conclusions. The discussion places the findings in context with existing literature, though a deeper critical analysis of limitations and more references to support claims would enhance the robustness of the interpretation. Overall, the sections are convincing but could benefit from additional clarity and a more thorough reflection on study limitations and future directions.

**Figures:**

The figures in the manuscript do not provide sufficient clarity, which hinders their effectiveness in conveying the data. In research articles, figures play a crucial role in visualizing complex information, and they must be easily interpretable. Unfortunately, the current figures appear unclear, possibly due to low resolution or inadequate labeling. It is recommended to increase the resolution of the figures and ensure that all axes, legends, and data points are clearly labeled and easy to read. Additionally, consider using contrasting colors or more distinct markers to differentiate between data sets. Improving the overall quality and clarity of the figures will significantly enhance their ability to support the text and help readers better understand the findings.

**Tables:**

The design of the tables in the manuscript could be improved for better clarity and readability. Currently, the tables have unnecessary borders on the left, right, and inner sections, which clutter the presentation of data. It is recommended to remove the left and right borders, as well as the inner borders, to create a cleaner, more streamlined appearance. This would help focus the reader's attention on the content rather than the table structure, enhancing the overall visual presentation and ease of interpretation. Additionally, adjusting the spacing between columns and ensuring consistent font size and alignment across all tables would further improve their clarity.

**Grammar and Style:**

The manuscript contains several grammatical and stylistic issues that hinder its clarity and flow. The writing requires careful revision to improve sentence structure, punctuation, and word choice. Additionally, some phrases appear awkward or overly complex, which may confuse the reader. To enhance readability and ensure the paper meets academic standards, it is recommended that the manuscript be polished by a native English speaker or a professional editor. This will help refine the language and ensure the clarity of the ideas being presented.

**Recommendation:**
Minor Revision. Addressing these points will enhance the clarity and impact of the manuscript.

Top of Form

Bottom of Form
