# Supplementary material for: Mitochondrial genomics and phylogeny of noctuoid moths: Implications for Macroheterocera
Source: PLoS One. 2025 Oct 7;20(10):e0333540. doi: 10.1371/journal.pone.0333540 (PMC12503346; doi:10.1371/journal.pone.0333540)
Supplement: Attachment — Submitted filename: Response to Editor.docx [file pone.0333540.s011.docx]

**Response to Editor’s comment**

**Comment**: In your Methods section, please provide additional information regarding the permits you obtained for the work. Please ensure you have included the full name of the authority that approved the field site access and, if no permits were required, a brief statement explaining why.

**Response**: Based on the editor comment, permission details were included in the methods section of the manuscript.
